# Modulation of orientation-selective neurons by motion: when additive, when multiplicative?

**DOI:** 10.3389/fncom.2014.00067

**Published:** 2014-06-20

**Authors:** Torsten Lüdge, Robert Urbanczik, Walter Senn

**Affiliations:** Computational Neuroscience Group, Department of Physiology, University of BernBern, Switzerland

**Keywords:** motion, gain modulation, additive modulation, contour detection, V1, illusions, model

## Abstract

The recurrent interaction among orientation-selective neurons in the primary visual cortex (V1) is suited to enhance contours in a noisy visual scene. Motion is known to have a strong pop-up effect in perceiving contours, but how motion-sensitive neurons in V1 support contour detection remains vastly elusive. Here we suggest how the various types of motion-sensitive neurons observed in V1 should be wired together in a micro-circuitry to optimally extract contours in the visual scene. Motion-sensitive neurons can be selective about the direction of motion occurring at some spot or respond equally to all directions (pandirectional). We show that, in the light of figure-ground segregation, direction-selective motion neurons should additively modulate the corresponding orientation-selective neurons with preferred orientation orthogonal to the motion direction. In turn, to maximally enhance contours, pandirectional motion neurons should multiplicatively modulate all orientation-selective neurons with co-localized receptive fields. This multiplicative modulation amplifies the local V1-circuitry among co-aligned orientation-selective neurons for detecting elongated contours. We suggest that the additive modulation by direction-specific motion neurons is achieved through synaptic projections to the somatic region, and the multiplicative modulation by pandirectional motion neurons through projections to the apical region of orientation-specific pyramidal neurons. For the purpose of contour detection, the V1-intrinsic integration of motion information is advantageous over a downstream integration as it exploits the recurrent V1-circuitry designed for that task.

## 1. Introduction

Experimental evidence has revealed different types of orientation- and motion-selective neurons in the primary visual cortex (V1). About one third of macaque V1-neurons respond selectively to the direction of motion (direction-selective cells Bourne et al., 2002), while other cells respond to motion with weak or no direction selectivity (called pandirectional Albright, 1984), or respond just to flicker (de Haan et al., 2013). The different degrees of direction selectivities of motion sensitive neurons are preserved in the processing from V1 to V4 (Douglass and Strausfeld, 1996; Lu et al., 2010; An et al., 2012). While the connectivity pattern among orientation-selective neurons was shown to support the detection of co-aligned edges (Gilbert, 1992; Li, 1998; Hess et al., 2003), little is known about the recurrent wiring of motion-selective neurons (Sincich and Horton, 2005; An et al., 2012), and even less about how motion- and orientation-selective neurons interact in V1.

The abundance of motion sensitive neurons in the visual cortex with their different types of selectivities raises the question of how they are involved in cortical computations. Motion processing so far has mainly been described as a feedforwad scheme that extracts motion information in a visual scene *per se*, and many of the classical orientation-selective neurons have been shown to also be sensitive to motion (An et al., 2012). Yet, motion sensitive neurons are also useful in analyzing a single snap shot of a sequence of frames. Detecting contours for figure-ground separation is such an operation that profits from combining orientation and motion information. How this information must be cross-combined on a computational level to best perform contour detection, and how this is implemented in the neuronal substrate, however, remains elusive.

Orientation- and direction-selective neurons, both represent cues for co-aligned edges. Directed motion detection is only possible perpendicular to an observed edge (the aperture problem), and hence direction-specific motion neurons are also informative about the existence of a perpendicular edge. This is suggestive to additively combine these types of cells for the sake of edge detection. Less obvious is whether pandirectional motion-sensitive neurons that do not carry directional information may support edge and contour detection. Here we show that this is still possible, provided that these pandirectional motion cells modulate the gain of all orientation-selective neurons that have their receptive field at the same narrow spot as the motion cell.

Neuronal modulation can arise, for example, from synaptic input targeting the soma of a cell, resulting in a shift of the neuronal response function (additive modulation). The modulation can also be multiplicative when excitatory synaptic input impinges the apical dendritic tree of pyramidal neurons, inducing a gain increase of the somatic response function (Larkum et al., 2004). While evidence for additive and multiplicative modulation was found for attentional signals in the visual cortex (McAdams and Reid, 2000; Thiele et al., 2009), additive modulation was shown to have drawbacks over multiplicative modulation in the context of contour integration (Schinkel et al., 2006). As we show, both the additive and multiplicative modulation by motion are computationally advantageous, depending on whether the motion-sensitive neurons are directional or non-directional.

Models of visual segmentation in V1 have a long history. The basic V1 circuitry underlying our and other models is inspired by Li (1999). Additional top-down modulations have been introduced to these models that locally enhance the neuronal gain (Schäfer et al., 2007; Piëch et al., 2013). This modulation acts as a local attentional signal that strengthens the perceived local image contrast and explains popup-effects. V1-models have also been endowed with additional long-range lateral connections to explain perceptual grouping (Grossberg and Raizada, 2000; Zhaoping et al., 2003). Instead of a top-down induction, the local modulation of the V1 neurons in our case is induced by motion signals that are extracted in V1 within or around the receptive fields of the co-localized orientation-selective neurons.

**Figure 1:**
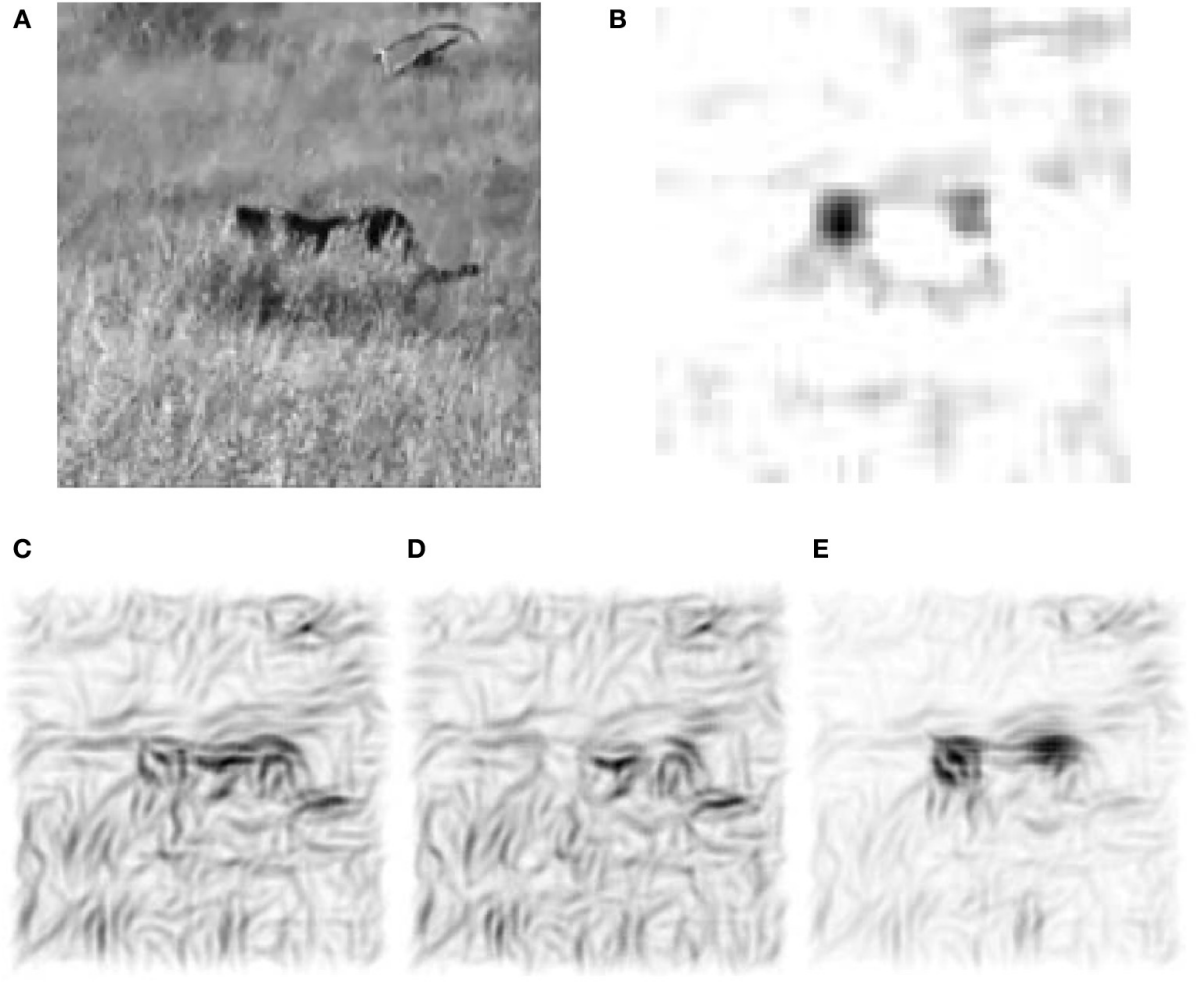
**Motion selective neurons in V1 improve figure-ground separation. (A)** Example frame from a natural movie. **(B)** Motion signal computed from the differences of frames showing the displacement of the animal as well as the waving grass. **(C)** Reconstruction with the Gabor-filter bank (see Methods) from V1 activities of the recurrent network that is not modulated by the local motion signals. (**D**) Stimulus reconstruction based on the orientation-selective V1 neurons that are additively modulated by orientation-unspecific motion signals. Via recurrent inhibition this merely hides the orientation information around the head region of the animal. **(E)** The reconstruction after multiplicative modulation by orientation-unspecific motion signals highlights the moving animal, even if the motion signal does not carry orientation information. This is achieved by a local gain increase that sharpens the contour extraction through the recurrent connectivity.

The benefit of local motion modulation in interpreting static images can readily be exemplified by considering the V1-processing of a real-world scene with and without this modulation. In natural images, objects are partially covering each other, and animals to be detected are camouflaged by their skin mimicking the surrounding structures (Figure 1A). Yet, a slight movement of an animal in the scene causes a visual pop-up effect that greatly facilitates the figure-ground separation. How the detection task can be instantaneously solved if the neuronal circuitry is provided by motion information remains an open question. Our V1-model represents a first step to address this problem. The static frame-by-frame processing of the visual information by the V1-circuitry of orientation-selective neurons provides a poor basis for segregating objects from the for- and background (Figure 1C). Motion information, even when not providing information about the particular direction of the motion, yields a cue where an object is (Figure 1B). Yet, simply “overlaying” the orientation and motion maps, as this is achieved by an additive combination, would reduce the local contrast via feedback inhibition (Figure 1D). Only when the gain of all orientation-specific neurons at the location of motion is increased will the contour information be sharpened (Figure 1E). Using synthetic stimuli, we analyze the conditions when directed and non-directed motion signals in V1 optimally support contour detection in a noisy scene. This provides a testable prediction for the synaptic connectivity pattern from motion- to orientation-selective neurons in V1.

## 2. Materials and methods

### 2.1. Model-architecture

We consider rate-coded model neurons of the Wilson-Cowan type where the feed-forward input currents are assumed to originate from retinal projections via the lateral geniculate nucleus onto V1 pyramidal neurons with Gabor-like receptive fields. We neglect the various coordinate transformations and identify retinal coordinates with the image coordinates ξ = (ξ_1_, ξ_2_) with ξ ∈ {1, …, *N*_retina_}^2^ to describe the internal representation of the image. From the two-dimensional grayscale image-sequence *S*(ξ, *t*) local edge-orientations are extracted in the form of a feedforward current *I*^𝕗^(*x*(ξ), θ, *t*) driving the θ-orientation selective V1 neurons at position *x*(ξ) of the cortical sheet. This is achieved by spatial convolution of the input image with a Gabor-kernel, centered at the 2-dimensional cortical coordinate *x* = (*x*_1_, *x*_2_) with *x* ∈ {1, …, *N*_V1_}^2^,

$$\begin{array}{l} G_{x\theta }(\xi )=\{\begin{array}{l} \exp\left(-\frac{{\left(\xi_{1}^{\theta }-{\tilde{x}}_{1}\right)}^{2}+\beta^{2}{\left(\xi_{2}^{\theta }-{\tilde{x}}_{2}\right)}^{2}}{2\sigma_{G}^{2}}\right)\cdot \cos\left(2\pi \frac{\xi_{2}^{\theta }}{\lambda }\right)\\ \begin{array}{ll} & \text{if }\max\{|{\tilde{x}}_{1}-\xi_{1}|,|{\tilde{x}}_{2}-\xi_{2}|\}\leq \sigma_{\text{RF}}\\ 0 & \text{otherwise} \end{array} \end{array}\\ \;\xi_{1}^{\theta }=\xi_{1}\cos\theta +\xi_{2}\sin\theta \\ \;\xi_{2}^{\theta }=-\xi_{1}\sin\theta +\xi_{2}\cos\theta \\ \;\tilde{x}=\sigma_{\text{RF}}x-\left\lfloor \sigma_{\text{RF}}2\right\rfloor \;, \end{array}$$

where ⌊·⌋ denotes flooring, θ ∈ {0°, 45°, 90°, 135°} are the equally spaced preferred orientations, σ_*G*_ = 51 and $\beta =\frac{1}{4}\sigma_{\text{G}}$. Hence, *I*^𝕗^(*x*, θ, *t*) = *I*^𝕗^_*x*θ_(*t*) = ∑_ξ_*G*_*x*θ_(ξ)*S*(ξ, *t*) constitutes the V1 feed-forward currents in cortical coordinates. Since the receptive fields are non-overlapping the convoluted image is of much lower spatial resolution than the retinal image (*N*_retina_ = σ_RF_*N*_V1_, *N*_V1_ = 15, σ_RF_ = 61).

The recurrent input is defined through the interaction of each orientation-selective neuron with its closely surrounding neighbors depending on their orientation and location. Similarly constructed as in Chisum et al. (2003); Ernst et al. (2012), the association field mimics experimental findings of co-alignment facilitation (Stettler et al., 2002; Bock et al., 2011) and cross-orientation inhibition (Priebe and Ferster, 2006). The contribution of surrounding neurons is calculated via the connection matrix *W*. We construct the balanced matrix *W*^*x*′θ′^_*x*θ_ = *w*_0_*R*(*x*, *x*′)*A*(θ, θ′, α) − ζ featuring excitation of co-aligned orientations, and surround suppression from an exponentially decaying radial part being independent of the preferred orientation θ at locations *x* and *x*′: *R*(*x*, *x*′) = *e*^−|*d*|^2^/2σ^2^^, $x^{\prime }\$:\$R(x,x^{\prime })=e^{-|d{|}^{2}2\sigma^{2}},\;d=\sqrt{{\left(x_{1}-{x^{\prime }}_{1}\right)}^{2}+{\left(x_{2}-{x^{\prime }}_{2}\right)}^{2}}$ and an angular part which contains the co-alignment prior: *A*(θ, θ′, α) = cos^2γ^ 2max[θ − θ′, α − θ, α − θ′] − *A*_0_, where α = arg((*x*_1_ − *x*′_1_) + *i*(*x*_2_ − *x*′_2_)) is the orientation of the line connecting the two locations *x* and *x*′. The skewing exponent γ narrows the excitatory range to opening angels smaller than 45° for γ > 1 and *A*_0_ ≥ 0 shifts the maximal inhibition (see Parameter optimization). We set ζ = 0.3 to normalize the net current per neuron to zero. The recurrent currents *I*^rec^ are computed via summation of the weighted, surrounding activities: *I*^rec^_*x*θ_ = ∑_*x*′θ′_*W*^*x*′θ′^_*x*θ_*r*_*x*′θ′_. The dynamics of the firing rates *r*_*x*θ_(*t*) is driven by a sigmoidal function $\rho \left(I\right)=\frac{1}{1+e^{-I}}$ of the total input current *I*. This current can itself be modulated by the motion signal *m*(*t*) in a multiplicative way (as the gain *g*_0_) or additive way (as the baseline firing rate *s*_0_),

(2) $$\begin{array}{l} \text{multiplicative}:\tau \frac{d}{dt}r_{x\theta }\left(t\right)=-r_{x\theta }\left(t\right)+\rho (\left(m(t)+g_{0}\right)\\ \left(I_{x\theta }^{\text{f​f}}\left(t\right)+I_{x\theta }^{\text{rec}}\left(t\right)-s_{0}\right)) \end{array}$$

(3) $$\begin{array}{l} \text{additive}:\tau \frac{d}{dt}r_{x\theta }\left(t\right)=-r_{x\theta }\left(t\right)+\rho (g_{0}(I_{x\theta }^{\text{f​f}}\left(t\right)+I_{x\theta }^{\text{rec}}\left(t\right)\\ +m\left(t\right)-s_{0})). \end{array}$$

**Figure 2:**
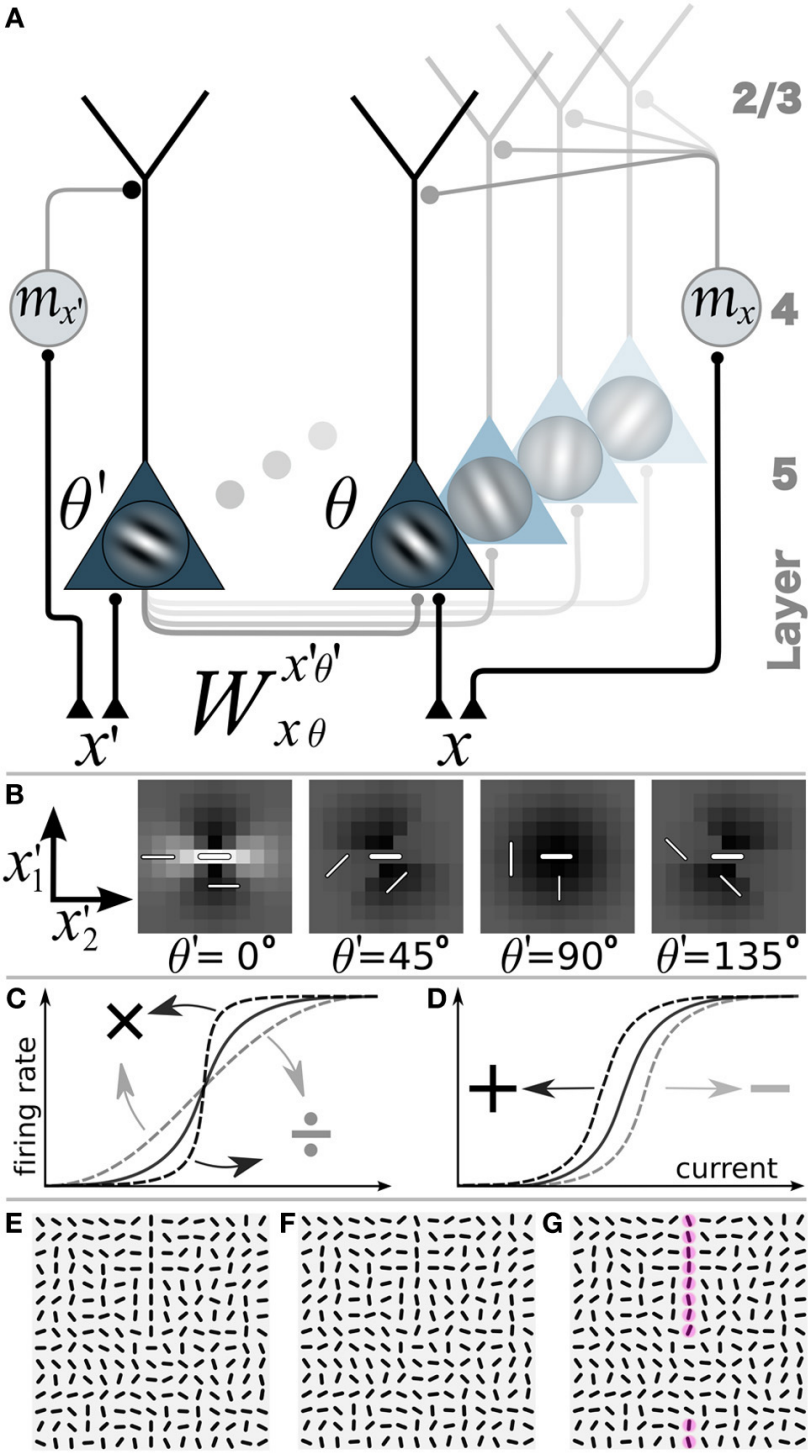
**Network model with gain-modulation and example stimuli**. **(A)** Sketch of the network composed of layer 5 orientation-selective pyramidal neurons (triangles, Gabor filter indicating preferred orientation θ). Motion sensitive cells (circle) project to the apical tree and multiplicatively modulate the transfer function of the pyramidal neurons (or project to the soma and shift the transfer function, not shown). A motion sensitive cell at cortical position *x* can convey a orientation-unspecific signal *m*_*x*_ or an orientation-specific signal (*m*_*x*,θ_, not shown). **(B**) Synaptic connection strengths *W*^*x*′, θ′^_(0,0),0_ of the neuron at position *x* = (0,0) and orientation-selectivity θ = 0° (central bar) to neurons with orientation selectivity θ′ = 0°, 45°, 90°, 135° at position (*x*′_1_, *x*′_2_). Bright: excitation, dark: inhibition. **(C)** Multiplicative or gain modulation of a pyramidal neuron transfer function. **(D)** Additive modulation. **(E)** Examples of stimuli presented to the retina with a vertical target-line (length = 10 segments, interrupted by 5 segments of different orientations) embedded in a background of randomly oriented orientations. **(F)** Stimulus with orientational noise from the interval [−12.5°, 12.5°] added to the vertical target-line. **(G)** Additional motion information represented by the red spots on the target line. Motion is modeled as a binary signal at a specific location that may or may not carry information about the orientation of the target line (Θ^T^).

The time constant of the neuronal dynamics is set to τ = 5 ms. We consider three different motion scenarios. In the static case without motion we set *m*(*t*) = 0 at all times and locations. If motion is present, the V1 motion signal may only depend on the position *x* in visual field but not on the direction of the motion at that position. In this case, the V1 motion signal is called direction-unspecific or pandirectional. Alternatively, the motion signal may also depend on the direction of the motion at position *x*, in which case we call the motion signal direction-specific (Figure 2),

(4) $$\text{direction-specific}:m(t)=\;m_{x\theta }(t)$$

(5) $$\text{direction-unspecific}:m(t)=\;m_{x}(t).$$

In all simulations we used periodic boundary conditions for the recurrent interactions.

### 2.2. Stimuli

The stimulus patterns are composed of identical, monochromatic bar-elements of length σ_*G*_ and width β, centered at the discrete (15× 15) grid locations at retinal coordinates $\tilde{x}$ and showing orientation $\varphi (\tilde{x})$. Formally, we set bar(ξ) = 1 if |ξ_1_| ≤ σ_*G*_ and |ξ_2_| ≤ β, and bar(ξ) = 0 otherwise, and defined the stimulus $S(\xi ,t)=\sum_{\tilde{x}}\text{bar}\left(\xi^{\varphi (\tilde{x})}-\tilde{x}\right)$, see Equation 1 for transformed ξ and Figures 2E–G. These stimuli contain one straight target-line with orientation Θ^T^ equal to one of the four cardinal orientations (i.e., with horizontal, vertical, diagonal orientations). A target-line has a total length of *N*_T_ = 10 co-aligned bar-elements. Due to the periodic boundary conditions this yields a gap of 5 bar elements with orientations strongly differing from the target-orientation Θ^T^ (deviation > 45°). The locations on the target-line in cortical coordinates are termed *x*_onT_ and locations away from the target-line are termed *x*_offT_. The orientation φ(*x*_onT_) of a bar on a target-line is jittered by an additive noise drawn from the uniform random distribution of width η_θ_ such that φ(*x*_onT_) ∈ [Θ^T^ − η_θ_/2, Θ^T^ + η_θ_/2]. Orientations φ(*x*_offT_) are uniformly drawn from the full interval [0°, 180°].

To define a motion signal we assume that the bars can move at constant velocity orthogonal to the orientation of the target-line Θ^T^. A motion-sensitive neuron centered at cortical location *x* that is direction-specific provides information about the presence of motion and the orientation of the target line via binary variable *m*_*x*θ_ = *m*_◦_ > 0 if θ = Θ^T^ and *m*_*x*θ_ = 0 if θ ≠ Θ^T^. If the motion-sensitive neuron is direction-unselective (pandirectional) it encodes only the presence (*m*_*x*_ = *m*_◦_ > 0) or absence (*m*_*x*_ = 0) of motion at that location. If motion is present in a stimulus pattern it is confined to all target locations (*m*_◦_) and absent (0) at all background locations. The specific value of *m*_◦_ is optimized for each scenario separately. In the pattern classification task (**Figure 6**) we additionally introduced a motion noise. The noisiness of the motion signal with noise parameter η_*m*_ ∈ [0, 1] is implemented for pandirectional cells by setting *m*_*x*_onT__ = 0 for η_*m*_*N*_T_ random target locations while *m*_*x*_offT__ = *m*_◦_ for the same number of background locations. Similarly, for directional cells we set *m*_*x*_onT_Θ^T^_ = 0 and *m*_*x*_offT_Θ^T^_ = *m*_◦_ for η_*m*_*N*_T_ randomly chosen on- and off-target locations, with a random orientation θ at each off-target location.

### 2.3. Orientation estimation and error measure

**Figure 3:**
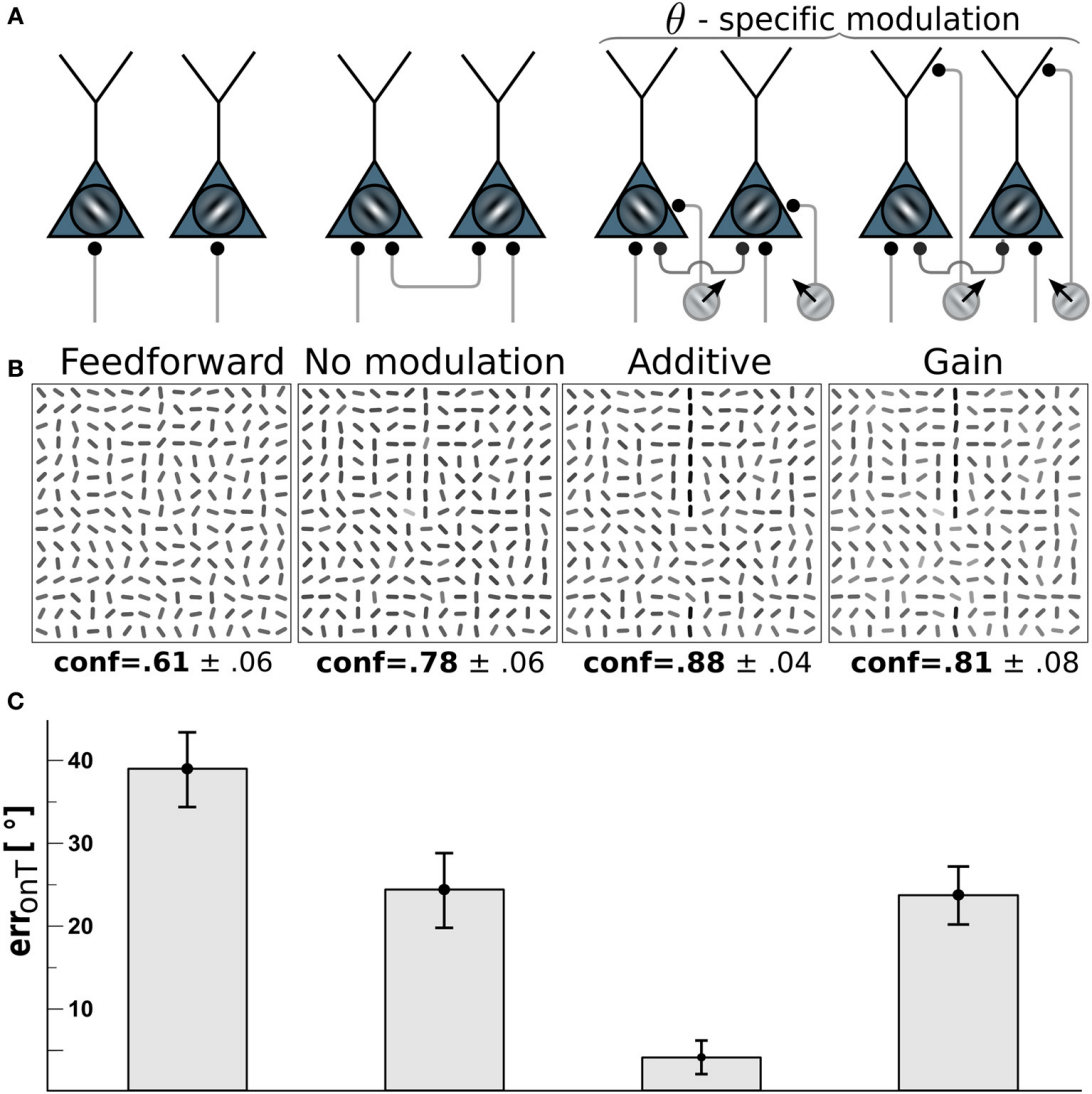
**Edge detection with direction-selective motion cells profits most from additive modulation**. (**A**) Network-wiring from left to right: feed-forward only, motion-free with lateral recurrent connections, recurrent network with direction-selective cells targeting the soma (additive) of orientation-selective cells with orientations orthogonal to the direction of motion, direction-selective cells targeting the dendritic tree (gain-modulation) of cells with orthogonal preferred orientation. (**B**) Reconstructions (as described in Orientation estimation) showing the orientations estimated from the V1 activity with wiring scheme depicted above. Gray-levels of the bar-elements represent the confidence of the estimated orientation. Numbers below: mean confidence about the true target orientation, spatially averaged across a target line and across the four cases of target locations. (**C**) On-target orientational error of the four scenarios. Error bars are standard error of the mean (s.e.m.).

The weighted sum of the steady state activities of all orientation-selective V1 units at one location in the complex plane *z*_*x*_ = ∑_θ_*r*_*x*θ_*e*^2iθ^ provides us with a good estimate of the presented orientation Θ^estim^_*x*_ = arg(*z*_*x*_)/2 at each location. The value of Θ^estim^_*x*_ then provides a measure for error correction by calculating its difference to the true orientation Θ^T^ of the presented (noise-free) target-line at each target location *x*_onT_. The on-target error across stimuli is computed as the mean difference to Θ^T^ at all locations along the target ${\text{err}}_{\text{onT}}(r)=\frac{1}{2N_{\text{T}}}\sum_{x_{\text{onT}}}\left|\arg\left(z_{x_{\text{onT}}}\right)-2\Theta^{\text{T}}\right|$. Similarly, the difference of the estimated orientations to the presented bar orientation φ at off-target locations ${\text{err}}_{\text{offT}}(r)=\frac{1}{2N_{\text{offT}}}\sum_{x_{\text{offT}}}|\arg\left(z_{x_{\text{offT}}}\right)-2\varphi_{x}|$ measures the distortion of the background. The average of the absolute values |*z*_*x*_| across target locations serves as a mean confidence measure of the estimated orientations along the target: ${\text{conf}}_{\text{ onT}}(r)=\frac{1}{N_{\text{T}}}\sum_{x_{\text{onT}}}\left|z_{x_{\text{onT}}}\right|$. The estimated orientation Θ^estim^_*x*_ is further used to reconstruct the stimulus *S*(ξ) with the same resolution as the input image through rotating either the bar elements (see Stimuli) or the Gabor filters (as for Figures 1C,D) by the orientation estimated at each location. Gray values of the rotated stimulus elements are scaled with the measured confidence value conf_x_(r) at each location *x*. The image reconstructed from the equilibrated V1 network state and weighted by the confidence values reveals its belief in local contours of the real-world stimulus (as seen for example in Figure 3B).

### 2.4. Parameter optimization

The width of the lateral connectivity σ effected the results only marginally and was chosen according to findings in Zipser et al. (1996) to have a half width of 3° and reach at most 10° which in our case corresponds to σ = 1.5 and a maximum interaction distance of 5 receptive fields to which the interaction *W* is truncated. The angular tuning parameter was set to γ = 4 as in Heitger et al. (1998); Kalar et al. (2010), and recurrent inhibition strength determined was set to *A*_0_ = 0.45 to match findings in Shushruth et al. (2013) where maximal suppression was found to reduce firing of the contextually modulated cell to about 50%. Concerning the maximal lateral excitation, *w*_0_, large variances have been reported by experiments ranging from 30 to 600% (Chisum and Fitzpatrick, 2004). Therefore *w*_0_, together with remaining parameters *g*_0_ and *s*_0_, were determined in a motion-free scenario, *m*(*t*) = 0, through minimization of the sum of the mean orientational error at on and off-target locations estimated from the neuronal activity **r** after processing our synthesized stimuli. This balanced measure err_Σ_(**r**) = err_onT_(**r**) + err_offT_(**r**) ensures that the parameters obtained from min_*w*_0_, *g*_0_, *s*_0__ err_Σ_(**r**) do not strongly distort the background representation while still correcting co-alignments in contours with low to intermediate orientation noise levels.

Results of the minimization were dependent on the orientational input noise-level η_θ_ (see Stimuli) which was set to the level at which the relative on-target error-correction η/err_T_ was highest (η_θ_ = 25°). The obtained slope of the transfer-function *g*_0_ = 3 is in agreement with the value used in a similar network described in Herzog et al. (2003) as is the value of *s*_0_ = 0.08 which gives a spontaneous firing of 8% of the maximal firing rate (Ringach et al., 2002). The magnitude of the motion signal *m*_*x*_(*t*) and *m*_*x*θ_(*t*) in the additive and multiplicative scenario was then adjusted to minimize the on-target error.

### 2.5. Classification-task

We used the perceptron algorithm on a training set of 2000 images, half of which contain a single prolonged line composed of 10 consecutive elements of orientation Θ^T^ (yielding a gap of 5 elements, see example in Figure 2F) jittered with a noise-level of η = 25° (see Stimuli). The other half is composed solely of randomly oriented bars. The motion signal along the target line exhibits a noisiness of 30% (η_*m*_ = 0.3, see Stimuli) and is copied to the 2nd class of stimuli not containing the contour line in order to not convey any information about the stimulus class in the motion channel. As before, the values of the binary motion signal *m*_*x*_ and *m*_*x*θ_ was adjusted in each wiring scenario to yield best classification performance. The synaptic strengths from the V1 neurons to the perceptron are adapted according to the perceptron learning rule, with the goal to distinguish the patterns with from the patterns without a target-line. Learning was stopped when the performance saturated. The learning-rate was optimized such that for half as well as 1.5 times the learning rate, convergence speed did not increase. The classification error given the neural responses elicited from a novel test set of 200 presented images (again half targets, half non-targets) are averaged over 15 trails with random initialization and compared for the following six scenarios: (1) pure feed-forward, (2) unmodulated recurrent, (3) additively, and (4) multiplicatively modulated recurrent network with pandirectional motion neurons, (5) additively, and (6) multiplicatively modulated recurrent network with directional motion neurons. As a control task, we also considered the classification based on parallel inputs to the perceptron from the unmodulated V1 activity and the separate motion signal.

## 3. Results

### 3.1. Modulation by direction-selective motion cells should be additive

To investigate which type of motion-orientation interaction yields the best performance in detecting elongated lines we considered stimuli composed of oriented bar elements. The performance is quantified by the error in reconstructing the true orientation Θ^T^ of the elements forming the target line. The reconstruction is based on the activities of the orientation-selective V1-neurons. We first considered the modulation of the V1-neurons by motion-sensitive cells that are also direction-selective. We assumed that a motion-sensitive cell projects to the co-localized orientation-selective neuron with preferred orientation orthogonal to the preferred motion direction. The motion signal *m* in Equation 3 in this case becomes specific to the preferred orientation θ at location *x*, hence, *m* = *m*_*x*θ_(*t*). We compared the two alternative wiring scenarios where direction-selective cells either project to the soma of orientation-selective cells, thereby additively modulating their activity, or project to the dendritic region and modulate their activity multiplicatively (see Methods and Figure 3A). For both cases the residual orientational error along the target, err^onT^(**r**), is compared to the baseline performance of the unmodulated, recurrent network with the uniform motion signal *m* = 0.

Because we assume that motion-sensitive cells also carry orientation information, the motion on the target line helps to detect the underlying orientation of the line. To keep the difficulty of the task, we considered a high orientation noise level of η = 40° on the bar elements forming the target lines. This 40° orientation error remains present in the reconstruction of the stimulus based on the feedforward activation of the V1 orientation-selective neurons (Figure 3, 1st column). When turning on the recurrent connections among the orientation-selective neurons, the reconstructed stimulus tends to co-align the bar elements and the orientation error on the target line is reduced to roughly 25° (Figure 3, 2nd column). When further considering the additive modulation by the direction-selective motion cells, the reconstruction error fell below 5° (Figure 3, 3rd column).

Interestingly, multiplicative modulation by the direction-selective motion cells did not improve the performance beyond the level of the recurrent, unmodulated network (Figure 3, last column). The reason why orientation-specific gain modulation has limited impact is the gain increase in weakly activated Θ^T^-selective neurons located on the target may actually reduce the activity of these line-representing neurons (see Figure 2C). In contrast, when the direction-selective motion cells additively drive the orientation-selective neurons, the activity of the Θ^T^-selective on-target neurons always increases (see Figure 2D). This supports the intuition that evidences about the presence of a specific orientation at a target location should be additively combined, not multiplicatively. In the present case, there are conditionally independent evidences about Θ^T^ deriving from the motion-sensitive and the motion-insensitive neurons that need to be added, not multiplied.

### 3.2. Modulation by pandirectional motion should be multiplicative

**Figure 4:**
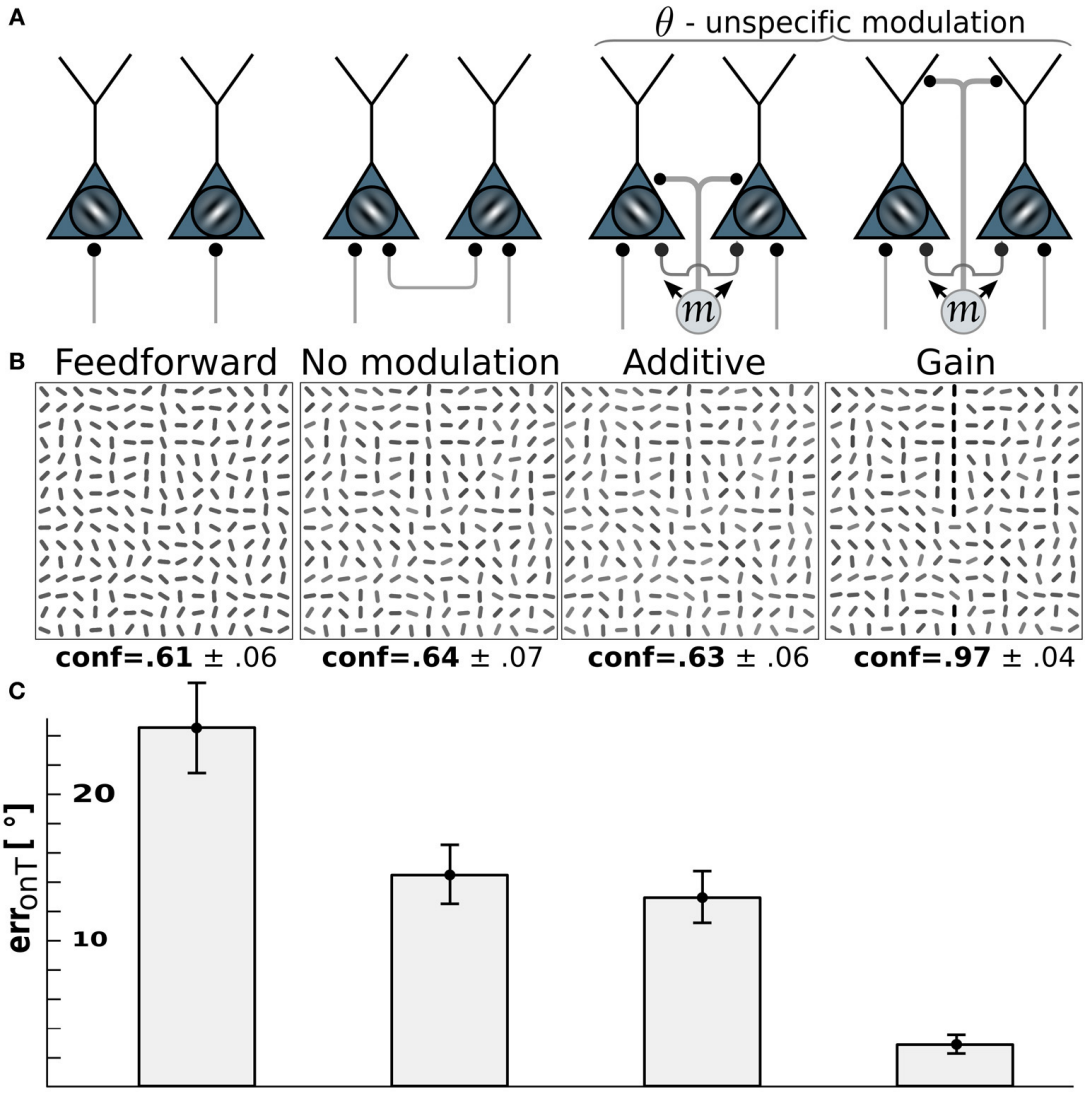
**Edge detection with pandirectional motion cells profits most from gain-modulation**. **(A)** Wiring scenarios. 1st and 2nd column from left as in Figure 3. 3rd column: additive modulation of all orientation-selective neurons at a given location by pandirectional motion cells at the same location. 4th column: as in column 3, but multiplicative modulation. (**B**) Reconstructions of the network activity in the wiring scenario depicted above for the same stimulus example. (**C**) On-target orientation error of the corresponding scenario (as in Figure 3).

For motion-sensitive cells which either respond to motion in any direction (pandirectional) or to flicker, there is no preference for a wiring to specific orientation-selective cells. Consequentially, we assume a modulative influence of our pandirectional motion detectors to all orientation-selective neurons likewise. We compare the same modulation scenarios as before (additive vs. multiplicative). Because the line-detection task is now more difficult with the reduced information of the motion-sensitive cells, we decreased the orientation-noise level to η = 25°. This noise level is again reflected in the on-target reconstruction error for the case that the θ-neurons are only driven through the feedforward connections (Figure 4, 1st column). While turning on the recurrent connections among the orientation-selective neurons in V1 decreases the orientation-reconstruction error on a target line (Figure 4, 2nd column), the additive modulation by the direction-unspecific motion cells does not further decrease the error (Figure 4, 3rd column). In contrast, the multiplicative modulation by the pandirectional motion cells now yields a strong reduction of the error to roughly 3° (Figure 4, last column).

The reason why multiplicative modulation for pandirectional motion neurons improves line-detection is that it sharpens the V1-intrinsic circuitry among the orientation-selective neurons. These recurrent connections are tuned to enhance the co-aligned orientation segments that potentially form a line, while they suppress by lateral inhibition the orientation segments that are nearly orthogonal to each other. The additive modulation with a motion signal that is blind to the orientation would merely shift the inputs to all orientation-selective neurons without sharpening the recurrent dynamics that enhances co-alignments.

### 3.3. Modulation separates activity distributions

**Figure 5:**
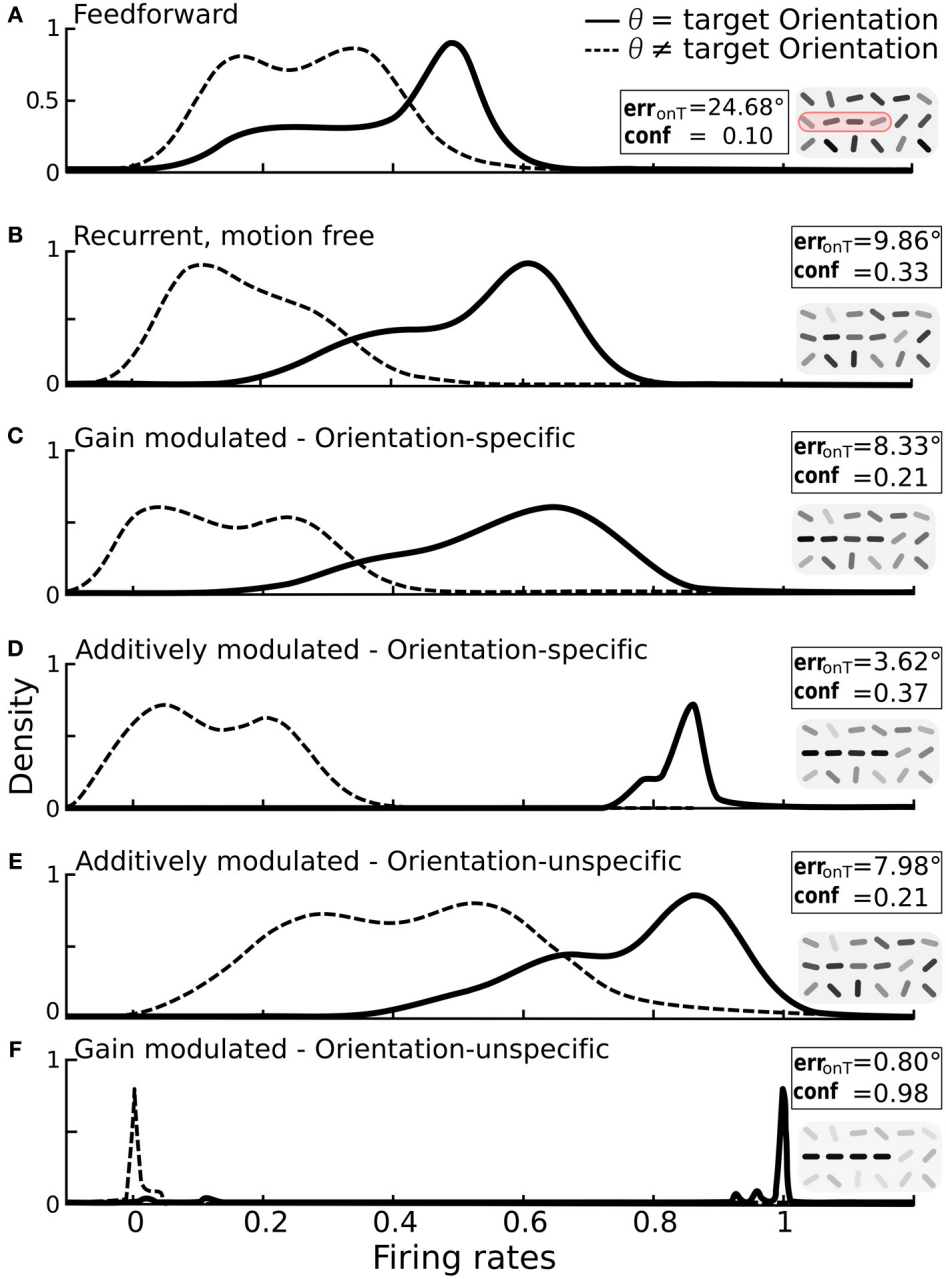
**Impact of additive vs. multiplicative modulation on the activity distributions of orientation-selective neurons at target locations**. For each scenario the same bar-stimuli with 25° orientational noise were used (see example in Figure 2F). Insets: on-target error and confidence values with reconstruction cutouts of the target region (gray-values are rescaled). **(A)** Solid line: distribution of firing rates for neurons at target locations with preferred orientation θ equal to the target orientation Θ^T^. Dashed line: distribution for neurons at target locations with preferred orientation different from the target orientation. With only feedforward connections, the two distributions strongly overlap. **(B)** Distributions after recurrent processing without motion-modulation i.e., *m*(*t*) = 0. **(C)** Multiplicative, orientation specific modulation by direction-selective motion (*m*_*x*θ_ = 1.4). **(D)** Additive, orientation specific modulation by direction-selective motion (*m*_*x*θ_ = 0.3). **(E)** Orientations-unspecific, additive modulation by pandirectional motion (*m*_*x*_ = 0.3). **(F)** Orientation unspecific, multiplicative modulation by pandirectional motion achieves a near-to-perfect separation of the two populations (*m*_*x*_ = 5). Histograms are smoothed using the kernel-density estimation method with a Gaussian kernel.

Orientation-tuning of V1 neurons can be broad. For our contour stimuli this would lead to overlapping activity distributions for neurons coding for the target orientations with those coding for non-target orientations. To explore the potential of the different network wirings in separating target from non-target activity we broadened the tuning curves of the edge-detectors until activities exhibited a strong overlap (Figure 5A). The distribution profiles when turning on the recurrent connections slightly separated the target orientations from non-target orientations (Figure 5B).

We again considered first the modulation by orientation-specific motion neurons. As expected, multiplicative modulation did only marginally improve the separation of activities from target and non-target neurons (Figure 5C). The broadening of the target activity profile shows that a gain increase of a target orientation can in fact decrease the activity if this is not large enough. This can appear when the orientation extracted by a motion neuron does not match the dominant orientation represented by the recurrent circuitry of orientation-specific neurons at that location. However, when the orientation-specific motion signal at a target location is added to the input of the corresponding orientation neuron, the activity always increases (Figure 5D).

For modulations with orientation-unspecific motion neurons the situation is reversed. Unspecifically adding the motion signal to the input of all orientation neurons at a target location increases the activities of all these neurons, irrespective of their assignment to target or non-target orientation (Figure 5E). The same unspecific motion signal acting multiplicatively, however, imposes a winner-takes-all mechanism among the orientation-specific neurons at a given location via cross-orientation inhibition and iso-orientation excitation. As consequence, the neurons are either driven to their maximal or minimal firing rates, and this typically reflects the correct assignment to the two classes (Figure 5F).

### 3.4. Classification of v1-activity patterns

**Figure 6:**
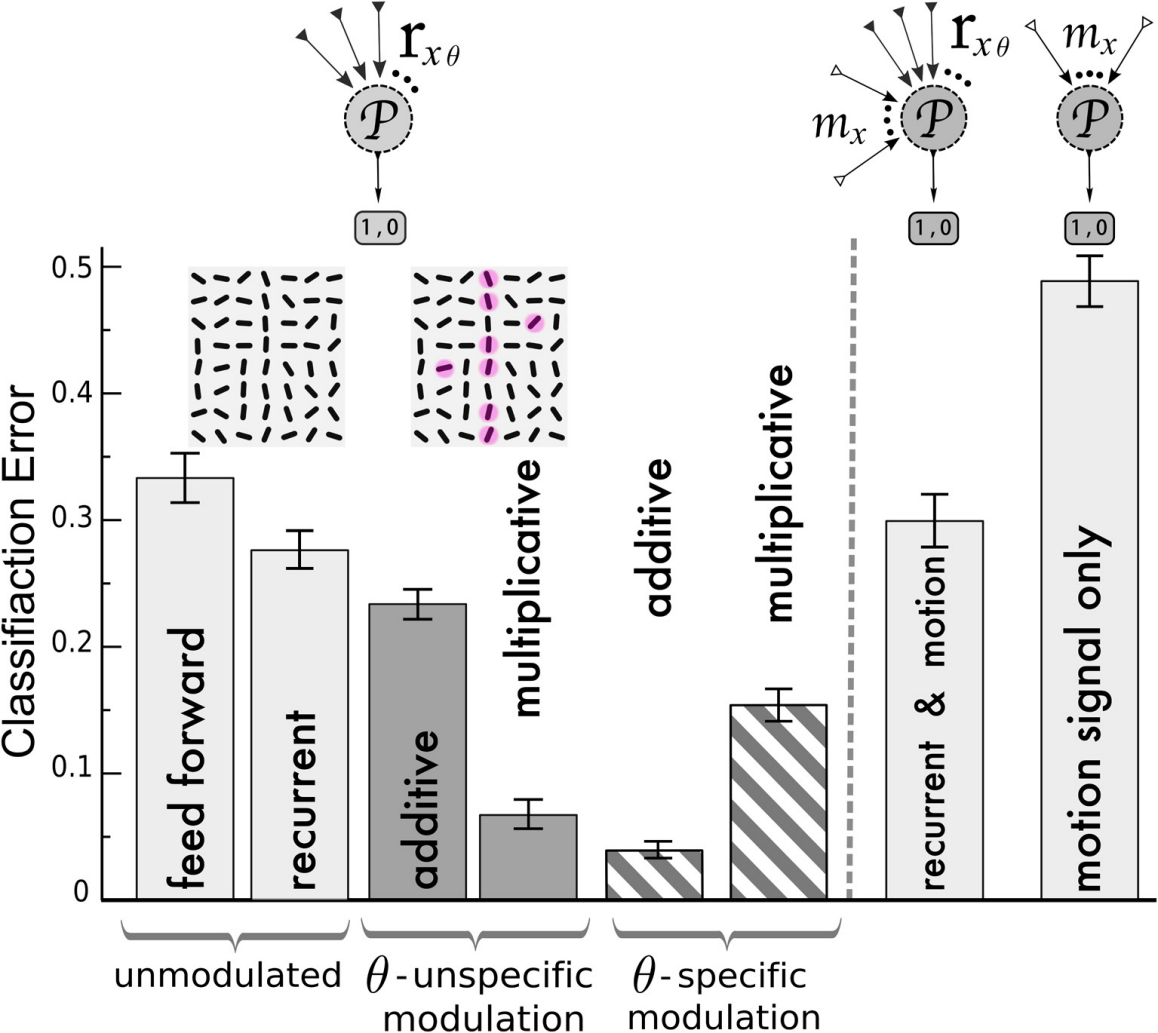
**Classification of V1 activity by a perceptron for stimuli with and without target lines**. Left of the dashed line, the perceptron (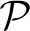) is fed by the activities *r*_*x*θ_ of the orientation-selective neurons after reaching the steady state of the neuronal dynamics. Classification error after training with the following network (from left to right): unmodulated feed-forward-only and recurrent recurrent (light gray bars); orientation-unspecific additive and multiplicative motion-modulation (dark gray bars, *m*^add^_*x*_ = 0.4, *m*^mult^_*x*_ = 3); orientation-specific motion modulation (striped bars, *m*^add^_*x*θ_ = 0.4, *m*^mult^_*x*θ_ = 1.3). Motion noise has been introduced to make the task more difficult. Insets: cutout of an example of a target stimulus, with red spots depicting motion (η_*m*_ = 0.3, see Methods). In agreement with the previous analysis, pandirectional motion neurons should multiplicatively and direction-specific motion neurons should additively modulate the orientation-specific V1 neurons to achieve best performances. Right of the dashed line: Classification error when the noisy motion signal *m*_*x*_ is not modulating the orientation-selective neurons but provided to the perceptron as an extra channel besides the *r*_*x*θ_ activities (2nd column from right) and when only the motion signal alone is provided (very right). Error bars represent standard deviations.

A central task of visual processing is to facilitate fast recognition of learned objects. As another test criterion for the contour enhancement capabilities of the two alternative modulations we therefore considered a classification of the V1 activity by a perceptron representing a readout in a downstream area. This binary classification task aims at correctly discerning two classes of patterns. One class consists of randomly oriented line segments (non-targets) whereas the second class contains as before a prolonged line with one of the four target orientations Θ^T^ and orientation-noise of 25° (target, example in Figure 6 inset).

We first compared the classification based on the responses of the orientation-selective neurons for the various modulation scenarios (Figure 6, left of dashed line): not modulated by motion (feedforward and recurrent) and modulated by an orientation-unspecific and a orientation-specific motion signal (each additive and multiplicative). For the feed-forward network, the classifier yields error-rates on novel test-sets at roughly one third (chance level 0.5). This performance is improved slightly by including the recurrenct connections without motion-modulation (2nd column), and it is again slightly improved by an unspecific additive modulation via pandirectional motion neurons (3rd column). Only when the pandirectional motion neurons modulate the local gain will the classification error significantly be reduced (4th column). In contrast, if the motion neurons are orientation-specific, it is the additive modulation that performs better (5th column) than the multiplicative (6th column).

As an alternative wiring of motion information we considered a direct projection of the motion neurons to a perceptron in a downstream area. The perceptron still receives input from the orientation-selective neurons in V1, but in this case these neurons were not modulated by motion (Figure 6, right of dashed line). Since we designed the motion signal to be present in the patterns of both classes, the motion information alone does not allow to discern these classes (Figure 6 very right column). Similarly, the motion input does not improve the classification when it is fed to the perceptron in parallel to the projections from the recurrently connected V1 network that was not modulated by motion-sensitive neurons (Figure 6 second column from right). As motion represents uninformative noise, learning is even slightly worse as compared to the case of the recurrent network without motion modulation (2nd column from left). In contrast, when the same motion signal modulates the gain, a putative target line will be enhanced and the classification is facilitated (Figure 6, 4th column from left).

### 3.5. Illusory contours

As visual illusions provide valuable insights into the machinery of visual processing (see Eagleman, 2001; Murray and Herrmann, 2013 and the references therein), we investigated the effects of the two network scenarios (additive vs. multiplicative modulation by pandirectional motion) when presented with static and moving illusory contours as seen in the Kanizsa illusion. Various experiments measured activity in the early visual areas that signal illusory contours in monkey (Von der Heydt et al., 1984; Grosof et al., 1993; Lee and Nguyen, 2001) and human V1 (Seghier et al., 2000). Those responses were found to increase when the stimulus is in motion (Seghier et al., 2000; Ni et al., 2003).

**Figure 7:**
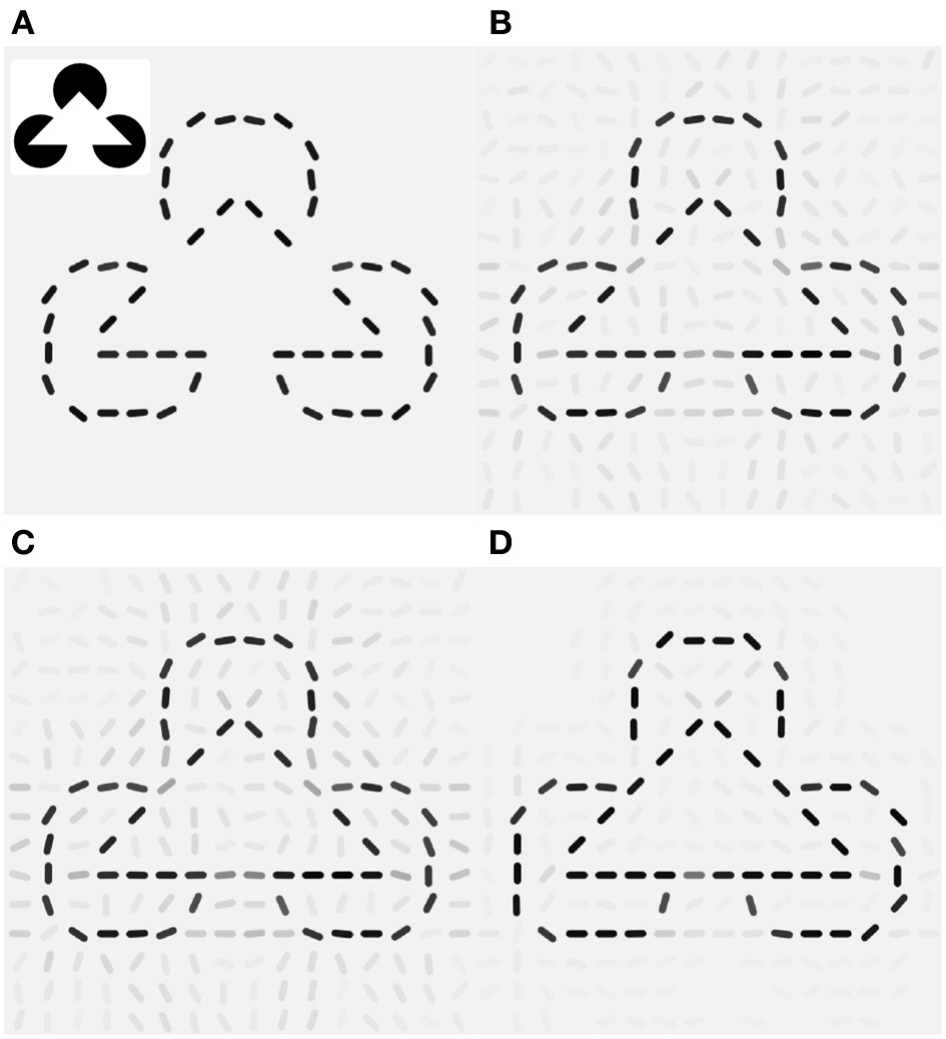
**Local gain-modulation generates illusory contours for moving Kanizsa stimuli**. (**A**) Reconstruction of the Kanizsa illusion (inset) from feed-forward activity. (**B**) Recurrent processing already exhibits responses of neurons with matching preferred-orientation at gap-locations along the triangles. Gray levels represent confidence values (see Methods). (**C**) Additive modulation by pandirectional motion cells increases responses but does not correct toward the illusory orientations. (**D**) Gain modulation increases not only signaling of the corresponding illusory orientation but also suppresses activities at locations with no neighboring contours and completes the illusory triangle with the matching orientations.

Since V1 neurons are unresponsive for the filled area of the pac-man discs the reconstruction of the original Kanizsa stimulus from the feed forward activity shows only the contour information (Figure 7A). The laterally connected V1 network is able to complete the gap in the illusory triangle, but it also signals spurious edges at other locations around the real contours (Figure 7B). Modulation by additive (orientation unspecific) motion does not increase the illusory percept (Figure 7C), only if the same pandirectional motion signal is acting multiplicatively a strong clean up effect is observed, eliminating most of the spurious edges (Figure 7D). Moreover gain-modulation corrected the estimated orientations at gap locations toward the orientation matching the illusory contour and increased their confidence level. Note that no end-stopping or bipolar mechanisms are implemented, therefore allowing some spread of activity around the triangle corners.

## 4. Discussion

We have shown how contour detection by the recurrent V1 circuitry can profit from motion information. Motion is extracted by neurons with different degrees of direction-specificity for motion (De Valois et al., 1982; Hawken et al., 1988). To support contour extraction, orientation-selective neurons should be differently modulated by these different motion sensitive neurons. When the direction selectivity is sharp, the motion neuron provides not only information about the motion direction itself, but also about the existence of an edge that is orthogonal to that direction. In fact, only when there is such an edge in its receptive field is a V1 neuron able to tell about motion. But many motion sensitive V1 neurons respond equally strong to moving edges that have different orientations, and hence these pandirectional motion neurons do not carry information about a specific orientation (Bourne et al., 2002; An et al., 2012). Yet, as we show, they may still support the extraction of oriented contours.

We have investigated different scenarios of how orientation-specific neurons in V1 are optimally modulated by motion-sensitive neurons for the sake of contour integration. We found that direction-selective neurons should additively modulate the corresponding orientation-selective cells, while pandirectional neurons should multiplicatively modulate all the orientation-specific cells at the spot of motion. The requirement for additive modulation arises from the fact that evidence about the existence of an orientated edge in the receptive field should be added, not multiplied. Hence, all neurons that carry information about a specific orientation should be additively combined, weighted by the corresponding degree of evidence. Biophysically, this can be achieved by synaptic projections from the direction-specific motion neurons to the somatic region of orientation-specific cells.

Pandirectional motion neurons that do not carry orientation information, instead, may act as a saliency signal, akin to attention, but narrowly localized to the receptive field of the motion neuron. As the underlying V1-circuitry among orientation-selective neurons is wired up to extract contours (Gilbert, 1992; Li, 1998), enhancing the gain of all these co-localized orientation-specific neurons will also enhance the extracted contour. This way, both the cooperativity among co-aligned orientation-selective neurons, and the competition among not aligned orientation-selective neurons, is strengthened. We suggest that this local gain modulation is achieved by synaptic projections to the apical region of pyramidal neurons that may display dendritic calcium spikes (Larkum et al., 2004).

Motion sensitive neurons with different degrees of direction selectivities are found in layer 3, 4, and 6 of V1 (Bourne et al., 2002; Gur and Snodderly, 2007), from where they may modulate the co-localized orientation-specific neurons. But the motion sensitive neurons may also be located in a higher visual area such as MT (Zeki, 1980; Albright, 1984; Felleman and Kaas, 1984) and project back to the orientation-selective neurons in V1 (Johnson and Burkhalter, 1996; Dong et al., 2004). As most of the motion neurons in the higher cortical areas are direction-selective (Baker et al., 1981; Maunsell and Van Essen, 1983; Felleman and Kaas, 1984), we predict that they are specifically targeting the corresponding orientation-selective neurons in V1. In fact, due to the larger receptive field, these motion neurons convey more reliable information about the true motion direction of an object contour. These top-down connections may become specifically wired up to match the corresponding orientations through Hebbian-type synaptic plasticity (Schäfer et al., 2007).

According to the classical view, motion and orientation information are represented in separated streams across the visual areas (An et al., 2012). This view implicitly assumes a feedforward combination of these information streams, for instance for classifying objects. However, when scenes have to be evaluated with regard to moving contours, our classification experiments show that the nonlinear interaction of motion and orientation within V1 pays out over a linear combination in a downstream area. These interactions may also lead to perceptual illusions when the scene violates the natural correlation statistics between motion and contours, as exemplified by the moving Kanizsa triangle (Ni et al., 2003). Since our model reproduces this illusion by the multiplicative gain modulation, we expect it to be also reflected in the V1 activity *in vivo*, similarly as illusory motion has been measured in V2 (Grosof et al., 1993; Lee and Nguyen, 2001). This would represent strong evidence for the suggested contour detection in V1 based on the motion-induced modulation of orientation-selective neurons.

One may speculate that the distinction between additive and multiplicative modulation also applies to the cortical representation of other sensory features or modalities. Whenever the modulatory signal carries the same specificity as the main signal, it should be additively combined. This is because evidence about the existence of that feature adds up. But if the modulatory signal carries less information about a feature, adding this information would merely blur the signal. A multiplicative modulation, instead, is expected to amplify the signal while preserving its feature specificity. The amplification may be further boosted by recurrent connectivity that sharpens the representation of that feature. Given the multiplicative and additive operation of apical and proximal synaptic input to cortical pyramidal neurons, respectively (Larkum et al., 2004), this distinction in turn would imply that apical dendritic input to these neurons is less specific than proximal input.

### Conflict of interest statement

The authors declare that the research was conducted in the absence of any commercial or financial relationships that could be construed as a potential conflict of interest.
